# Touching to Feel: Brain Activity During In-Store Consumer Experience

**DOI:** 10.3389/fpsyg.2021.653011

**Published:** 2021-03-23

**Authors:** Michela Balconi, Irene Venturella, Roberta Sebastiani, Laura Angioletti

**Affiliations:** ^1^International Research Center for Cognitive Applied Neuroscience (IrcCAN), Catholic University of the Sacred Heart, Milan, Italy; ^2^Research Unit in Affective and Social Neuroscience, Department of Psychology, Catholic University of the Sacred Heart, Milan, Italy; ^3^Department of Economics and Business Management Sciences, Catholic University of the Sacred Heart, Milan, Italy

**Keywords:** EEG, touch, consumer experience, in-store research, wireless system, consumer awareness

## Abstract

To gain a deeper understanding of consumers' brain responses during a real-time in-store exploration could help retailers to get much closer to costumers' experience. To our knowledge, this is the first time the specific role of touch has been investigated by means of a neuroscientific approach during consumer in-store experience within the field of sensory marketing. This study explores the presence of distinct cortical brain oscillations in consumers' brain while navigating a store that provides a high level of sensory arousal and being allowed or not to touch products. A 16-channel wireless electroencephalogram (EEG) was applied to 23 healthy participants (mean age = 24.57 years, SD = 3.54), with interest in cosmetics but naive about the store explored. Subjects were assigned to two experimental conditions based on the chance of touching or not touching the products. Cortical oscillations were explored by means of power spectral analysis of the following frequency bands: delta, theta, alpha, and beta. Results highlighted the presence of delta, theta, and beta bands within the frontal brain regions during both sensory conditions. The absence of touch was experienced as a lack of perception that needs cognitive control, as reflected by Delta and Theta band left activation, whereas a right increase of Beta band for touch condition was associated with sustained awareness on the sensory experience. Overall, EEG cortical oscillations' functional meaning could help highlight the neurophysiological implicit responses to tactile conditions and the importance of touch integration in consumers' experience.

## Introduction

Aware that the consumer's behavior is the complex result of a multifaceted interaction between the organism and its environment (Holbrook and Hirschman, 1982), customer experience has been defined as “the aggregate of feelings, perceptions and attitudes formed during the entire process of decision-making and consumption chain […] leading to cognitive, emotional, sensorial and behavioral responses” (Jain et al., 2017) and can be grounded in the theory of organism response by Mehrabian and Russell (1974), for which the consumer's responses (i.e., approach or avoidance) are determined by the interaction between stimulus and organism (i.e., consumer's emotional state of pleasure, arousal, and dominance) (Mehrabian and Russell, 1974). The implicit processing underlying the interaction between stimulus and organism can be studied by means of neurophysiological tools applied during the consumer's experience; indeed, these tools can help to obtain relevant information on the ongoing covert sensory experience while touching products that are not directly achievable with classical self-report questionnaire or scale.

Within the frame of sensory marketing, previous research showed the importance of the sense of touch during the in-store consumer experience, considering both positive consequences and negative effects (i.e., tactile contamination) of touching products (Citrin et al., 2003; McCabe and Nowlis, 2003; Argo et al., 2006; Peck and Childers, 2006; Peck and Wiggins, 2006; Peck and Shu, 2009; Peck and Johnson, 2011). Indeed, the sense of touch has often been considered as a booster of the consumer's experience, able to predict the will of purchasing a good, and, nowadays, the absence of tactile stimulation (i.e., the inability to handle merchandise) has been identified as one of the most well-known obstacles of online Web shopping experience (McCabe and Nowlis, 2003) that must be replaced by other positive intervening factors as the promotion of a positive mood state and/or the use of a touch interface while surfing the e-commerce (Yazdanparast and Spears, 2013; Chung et al., 2018).

To determine individual differences related to the specific need of touching products, before, a “Need For Touch” scale (Peck and Childers, 2003a,b) has been designed, and it includes two different dimensions: one more instrumental, and the other one related to the compulsive and emotional components of touch. These individual differences have been argued to influence the impact of humans touching products and products touching products (Krishna, 2012). Indeed, touch has widely been considered strictly related to emotion domain, given that physiologically, even skin surface is dedicated to the affective response coding (e.g., C Tactile nerve fibers; Spence and Gallace, 2011). Spence and Gallace (2011) argued that touch is also likely to provide “a less noisy estimate of a product's hedonic value” than other senses, and, accordingly, it has been highlighted that touch is connected to information and feelings on a product through physical and psychological interactions (Hultén, 2011).

However, there is still a lack of studies investigating the psychological dimensions and emotional aspects involved in sensory consumers' experience in-store by employing a neuroscientific approach and, in particular, touch is the least studied sense in the neuromarketing field. Nevertheless, we agree with previous research that this sense plays a key role in the emotional aspects involved during the sensory experience of the customer (Hultén, 2011, 2012, 2013; Klatzky, 2011).

For this reason, we conducted an empirical study applying electroencephalogram (EEG) to measure the involvement of senses, specifically touch, elicited by sensory cues during customers' store exploration. The use of an EEG wireless technology implies the capability to record brain waves at very small-time intervals, in the order of milliseconds, while consumers are exploring space. This is extremely valuable, considering the speed at which we acquire information through our senses and the speed of our thoughts (subseconds). Besides a good temporal resolution, within the neuromarketing field, the advantages of using EEG have been previously highlighted also by other scholars (Vecchiato et al., 2011); indeed, EEG wireless devices are portable, relatively low cost, robust, and suitable for evaluating marketing stimuli in an ecological environment if compared to other neuroscientific tools requiring a static setup (such as functional magnetic resonance imaging or magnetoencephalogram).

Overall, the aim of our study is to gain a deeper understanding of customers' neural activations related to emotional processing following exposure to certain sensory stimuli during an in-store exploration and to answer the call for papers launched in recent years within the field of sensory marketing asking for more impactful research (Krishna, 2012). Indeed, prior studies demonstrate that there is an impact of the retail space on the shopper's sensory and social stimulations, leaving the consumer pleasured and aroused during the shopping experience (Turley and Milliman, 2000).

Our experimental and extremely ecological setting was the inside of one store belonging to a popular cosmetics chain, well-known for the use of bright colors to stimulate sight, of high-volume pop music to stimulate hearing and positive feelings, and of perfumes deriving from the products rigorously exposed without packaging (characterizing the brand value aimed to provide a multisensory experience). In addition, customers are also given the chance to easily touch and try all the products exposed, thus providing a higher level of sensory arousal. With the purpose of exploring a single sense experience and the relative impossibility to use the others, selective sensory deprivation supports (earplugs for ear, plugs for the nose, and the instruction to “do not touch”) were applied on the person during a free shopping experience. The field of cosmetic was functional for exploring sensory integration/deprivation for two reasons: (i) the intrinsic features of cosmetic items are known to appeal to all five senses; (ii) if, during the analysis of a cosmetic product, consumers' senses are positively activated, this will possibly result in a positive appraisal of the perceived quality of the product and lead to an approach behavior due to the product's emotional connection (Theofanides and Kerasidou, 2012).

Specifically, cortical oscillations (EEG waves) observed during different conditions of sensory stimulation inside the store and their functional meaning were considered in order to understand the relevance of the presence and the absence of tactile stimulation in consumer experience. Within the neuroscientific Dual Systems model (Davidson, 1992) that connects emotional aspects and behavioral tendency to the anterior cerebral activation, a right frontal greater neural activity is associated with negative events, inhibitory control processing, and withdrawal-related behavior, while the presence of a left frontal neural activation reflects a positive emotional processing and an approach-related behavior.

According to this theoretical account and previous literature (Balconi and Mazza, 2010; Balconi and Bortolotti, 2012; Balconi et al., 2014a), we expected different neural oscillations based on ongoing emotional and cognitive processes while depriving or maintaining the sense of touch (Başar et al., 2001; Brovelli et al., 2004). In particular, for the non-touch condition – requiring subjects' behavioral adaptations to this deprivation –, it was hypothesized a poorer sensory experience, characterized by low frequency bands frontal left activation (Cavanagh and Shackman, 2015). On the other hand, it was supposed that the use of the sense of touch can be considered a positive condition with enhanced sensory processing reflected by higher-frequency band activation in sensorimotor brain regions. Moreover, the presence of unpredictable and possible compensatory neural mechanisms due to the isolation of a sense was considered.

## Materials and Methods

### Sample

A total of 23 Caucasian right-handed healthy participants were engaged in the experiment (five males; mean age = 24.57 years, SD = 3.54). Inclusion criteria were as follows: (1) interest in cosmetics, (2) not being a frequent customer of the store used as the experimental setting, and (3) normal or corrected-to-normal hearing and vision. Exclusion criteria were the presence of sensory and cognitive deficits, a history of psychiatric or neurological diseases, and ongoing concurrent therapies based on psychoactive drugs that can alter central nervous system functioning. No compensation was provided for their participation in the study. One participant was excluded from the statistical analysis due to the high presence of movement artifacts.

The study has been designed following the principles of the Declaration of Helsinki. Procedures and methods were approved by the Ethics Committee of the Department of Psychology, Catholic University of the Sacred Heart of Milan, Italy. Subjects gave written informed consent for their participation in the study.

### Procedure

Participants have been introduced in a neutral point of the store (warehouse), where non-invasive EEG sensors were placed, and then they were guided inside the store. Sensory deprivation supports were applied before starting the store exploration. Subjects were equally divided into two conditions based on the senses that subjects could use: (1) Touch (participants can use touch); (2) Non-Touch (participants can see, hear, and smell, with the instruction “do not touch”). The sight was kept as a constant to let participants explore the ecological setting (that is the store) freely. They were assigned to only one condition in order to avoid order and habituation effects. After the placement of the sensory deprivation supports, subjects were informed that they had time from a minimum of 5 min to a maximum of 15 min to explore the store freely, according to their experimental condition. During the whole experiment, EEG cortical activity was recorded.

### Electroencephalogram Recording and Neural Data Reduction

During store exploration, EEG activity was collected *via* an EEG wireless System (Live-Amp) and processed *via* Analyzer2 software (Brain Products GmbH, Gilching, Germany). The montage included 15 active electrodes (Fp1, Fp2, F3, Fz, F4, T7, C3, Cz, C4, T8, P3, Pz, P4, O1, O2; placement according to the 10-20 International System; Jasper, 1958). Electrode impedance was monitored for each subject prior to data collection and kept under 5 kΩ. Data were acquired using a sampling rate of 250 Hz and then filtered offline with a 0.5–45-Hz IIR bandpass filter (slope = 48 db/octave). Data were then segmented and visually inspected for ocular, muscle, and movement artifacts. Fast Fourier Transform (Hamming window, resolution = 0.5 Hz) was applied to artifact-free segments to compute the average power spectra. Finally, average power for the main EEG frequency bands (Delta = 0.5–3.5 Hz, Theta = 4–7.5 Hz, Alpha = 8–12.5 Hz, Beta = 13–30 Hz) were extracted (see Harmony, 2013; Balconi et al., 2019, for frequency bands range).

### Data Analysis

A set of mixed repeated measures ANOVAs with independent within-factors Region Of Interest (ROI) (3: Frontal [F3; F4], Central [C3; C4], and Parietal [P3; P4]) and Laterality (2: Left and Right) and as between factors the Condition related to sense of touch isolation or deprivation (2: Touch vs. Non-Touch) was applied on dependent EEG measures. This mixed repeated measures ANOVA was performed for each frequency band (Delta, Theta, Alpha, Beta) in order to highlight the differences between two conditions: the isolation of a sense (only the sense of touch allowed) and the deprivation of the same (only to touch was not allowed). *Post hoc* comparisons were applied to the data in case of significant effects. Simple effects for significant interactions were further checked *via* pairwise comparisons, and Bonferroni correction was used to reduce multiple comparisons potential biases. For all the ANOVA tests, the degrees of freedom have been corrected using Greenhouse–Geisser epsilon where appropriate. Furthermore, the normality of the data distribution was preliminarily assessed by checking kurtosis and asymmetry indices. The size of statistically significant effects has been estimated by computing partial eta squared (η^2^) indices.

## Results

### Delta and Theta Low-Frequency Bands

**Figure 1:**
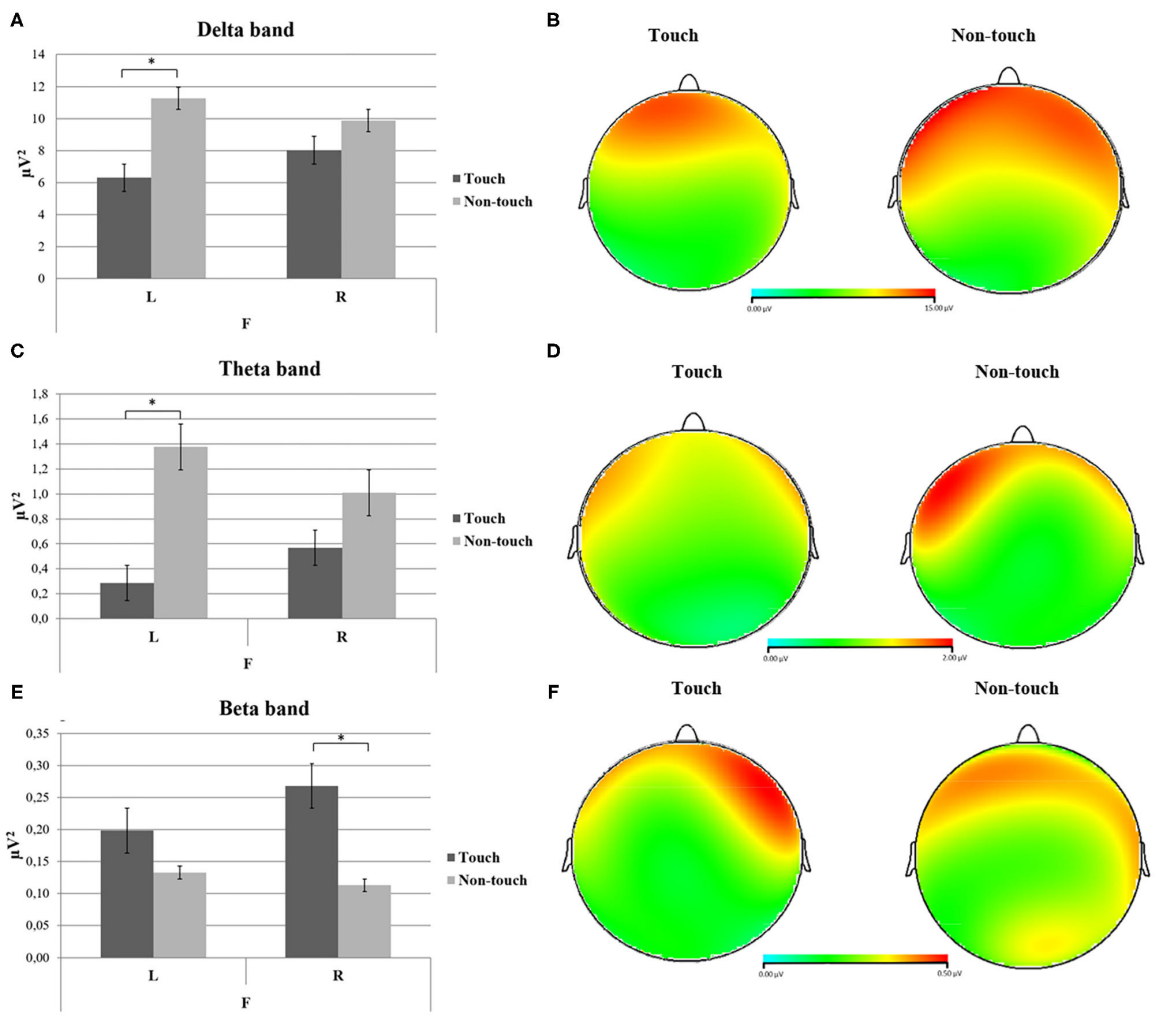
**(A–F)** Electroencephalogram (EEG) oscillatory activity (power values) over left and right frontal sides in response to Touch- and Non-touch-related conditions. **(A)** Bar graph shows significant differences for delta power in the left frontal area between touch and non-touch conditions. **(B)** Delta EEG power representation of touch condition (left head) compared to non-touch condition (right head). **(C)** Bar chart displays significant differences for theta power in the left frontal area between touch and non-touch conditions. **(D)** EEG theta power display for touch (left head) compared to non-touch condition (right head). **(E)** Bar chart shows significant differences for beta power in the right frontal area between touch and non-touch conditions. **(F)** Beta power EEG display for touch condition (left head) compared to non-touch condition (right head). For all bar charts, stars mark statistically significant pairwise comparisons, bars represent ±1 SE. For all EEG head displays, red represents the increase of power for each frequency band.

As shown by ANOVA for Delta band, interaction effect Condition × Laterality × ROI was found [*F*_(2, 22)_ = 5.49, *p* = 0.012, η^2^ = 0.33]. *Post hoc* pairwise comparisons revealed increased Delta power in left frontal area (F3) for the Non-Touch condition compared to the Touch condition [*F*_(1, 22)_ = 5.61, *p* = 0.037, η^2^ = 0.33] (Figures 1A,B). For Theta band, interaction effect Condition × Laterality × ROI was found [*F*_(2, 22)_ = 7.77, *p* = 0.004, η^2^ = 0.41]. *Post hoc* pairwise comparisons revealed increased Theta power in the left frontal area (F3) for the Non-Touch condition compared to the Touch condition [*F*_(1, 22)_ = 21.92, *p* = 0.001, η^2^ = 0.66] (Figures 1C,D).

### Beta Band

For Beta band, an interaction effect Condition × Laterality × ROI was found [*F*_(2, 22)_ = 3.82, *p* = 0.038, η^2^ = 0.25]. *Post hoc* pairwise comparisons revealed increased Beta power in the right frontal area (F4) for Touch condition compared to Non-Touch condition [*F*_(1, 22)_ = 5.11, *p* = 0.045, η^2^ = 0.31) (Figures 1E,F).

## Discussion

The aim of the present study was to explore customers' cortical response (brain oscillations) related to the presence and the absence of tactile experience during a free in-store navigation. Indeed, to isolate different conditions of sensory fruition helped us to deepen the knowledge on the role of the sense of touch in consumers' experience.

The performed frequency bands analysis contributed to underline the following main results connected to the role of frontal brain areas when cognitively processing the in-store exploration (in this case corresponding to the fruition of cosmetic products) with and without tactile sensory insights. Firstly, a higher presence of Delta and Theta band activity mainly lateralized on left frontal areas for the Non-Touch condition was found. In this condition, participants could explore the store freely using the senses of sight, hearing, and smell, yet with the instruction not to touch products. Secondly, an augmented Beta band power for the Touch condition was detected in the right anterior regions.

According to previous literature, this evidence can be discussed mainly focusing on the cognitive aspects related to the functional meaning of the detected frequency bands. Regarding our first results, the high presence of low-frequency bands in frontal anatomical structures could be considered mainly as a marker of cognitive operations involved during the situation in which a tactile exploration of products was not allowed. Indeed, starting from the cognitive features related to the manifestation of Theta band, previous studies identified that complex bimodal sensory stimulation increase the frontal processing in this band range (Başar et al., 2001). More broadly, an “orienting” function of this frequency band has been recognized, since its power was also observed in case of a coordinated response indicating alertness and readiness to process information during exploration, searching, and motor behavior (Başar, 1999); that is, theta EEG power typically increases with increasing attentional demands and/or task difficulty or uncertainty (Başar-Eroglu et al., 1992; Cavanagh and Shackman, 2015). In addition, Theta power also increases over mid- and lateral-frontal areas for events that involve a need for cognitive control, such as novel stimuli, conflicts, and errors (Cavanagh and Frank, 2014). Thus, one possible explanation could be that the experimental condition in which participants experienced the free exploration of the store without the chance to touch products, but maintaining the other multiple sensory stimulations, could have involved a sort of “sensory uncertainty” with the absence of the most salient sense (touch), resulting in an anomalous gestalt perception for the perceiver who was not able to gain a full perceptual understanding of the stimulus and, consequently, needs much more cognitive effort for processing it.

Conversely, limited data exist on the functional meaning of Delta band modulation and roughly similar to those mentioned for Theta oscillation, i.e., cognitive processing. In previous basic research by Başar-Eroglu et al. (1992), the amplitude of delta response was found to be considerably increased during oddball paradigms. And, accordingly, it has been concluded that Delta activity is related to signal detection and decision-making. In line with this, Knyazev (2007) showed that delta power depends on the activity of motivational systems and participate in stimulus salience detection (Knyazev, 2007). Moreover, Balconi et al. (2015) found that delta modulations were found to be related to arousing power of stimuli in right and left frontal localizations, regardless of the stimulus valence. Therefore, we concluded that, taken together, Theta and Delta increased activity may be responsive to a process of signal detection of the stimuli encountered during the store navigation; however, the absence of tactile contact could have given rise to a situation of incomplete environmental perception that alerted the consumers on the need for cognitive control on their experience. Further studies will need to explore the emotional meaning of the presence of Delta and Theta frequency bands in the left hemisphere during a condition of sensory deprivation.

Concerning our second result, a Beta band cortical pattern was found to be more lateralized on the right frontal hemisphere, suggesting a greater attentional focus on the touch condition that could have brought the subjects to a higher conscious activation. One possible explanation is that this experimental condition in which consumers are allowed to touch the products could have induced to an attentional activation mainly focused on the tactile and visual aspects of the product. Previous literature highlighted how frontal neural activity in the Beta band have been linked to sensorimotor network-enhanced activity together with the maintenance of the cognitive state (Brovelli et al., 2004). Regarding the functional role of Beta band activity during cognitive and perceptual processing, (Engel and Fries, 2010) determined that Beta power can be enhanced if there is the priority to maintain a cognitive state over potential new signals considered as distractive (Engel and Fries, 2010). More generally, a greater frontal right hemispheric activation was demonstrated to reflect also the inhibitory control processes (Garavan et al., 1999; Aron et al., 2014). Thus, it has been possible to suppose that, within our touch condition, a right Beta band activity in frontal areas is associated with sustained attention and maintenance of the cognitive set that overrides the effect of potentially novel, or unexpected, external events, providing an “augmented” and aware sensory and cognitive experience derived by the possibility to touch the products.

In addition, in evolutionary terms, the sense of touch (and haptic more in general) has ancestral roots and covers a central role within the sensory system, both phylogenetically and ontogenetically. Previous evolutionary studies show that even infant macaque monkeys prefer to physically approach a surrogate soft cloth mother (closer to their haptic representation of mother) than a wire mother (Harlow, 1958). Specifically, even if the wire mother provides nutrition, the cloth mother provides a warm and more coherent experience, thus being considered the favorite by the animal infants. This has been found to be true also for human infants, for which the physiological need for food and the instrumental role of the nutritive mother can be bypassed by the need for physical touch and effective contact (Montagu, 1971).

In the field of neuromarketing, the importance of touch, especially for clothing and fabric retail, has previously been recognized, and retailers use different marketing strategies aware and guided by the importance of touch (Citrin et al., 2003; McCabe and Nowlis, 2003). Indeed, some store chains make the displayed merchandise difficult to touch or, on the contrary, easy to touch on the basis or their marketing strategy. Our results stressed the idea that touch is a sense that should be maintained in retail strategies because it is able to provide the consumers a full and complete cognitive experience of the product, even when other senses are absent or temporarily isolated.

However, so far, no previous studies investigated the specific role of touch by means of EEG power spectral analysis within the field of sensory marketing, specifically when exploring cosmetic products. For this reason, future research will need to deepen our insights and investigate if they can be broadly transferred into the wide context of sensory marketing, in which we believe that touch can cover a discriminating role for consumers' experience exploring cosmetic products.

Moreover, further studies will be necessary to investigate the lateralization effect we found in relation to the different frequency bands and the possible specific role of Delta and Theta oscillations in left frontal structures as a possible marker of processing the emotional valence of consumers' experience. Indeed, frontal and prefrontal cortex lateralization has been previously related to cognitive control over emotional stimuli and emotional behavior in basic research and in studies on cross-modal integration of emotional cues (Balconi et al., 2015; Balconi and Vanutelli, 2016). In past neuromarketing studies, a left prefrontal cortex activation toward commercial advertising was interpreted as an index of positive emotions and consumers' preference (Balconi et al., 2014b; Leanza and Balconi, 2017).

So far, to our knowledge, there is only one basic research on the valence of tactile stimulation showing an increase in right temporoparietal and frontal electrodes in the beta range for pleasant products compared to unpleasant ones (Singh et al., 2014). This study suggested the possible usefulness of the Beta band to more directly measure affective states and higher-order cognitive sensory aspects in a wide range of areas of interest where touch is involved, such as neuromarketing and consumer research (Solnais et al., 2013). Since these previous studies have been applied in different contexts from the cosmetic field, caution is needed in affirming that touch and non-touch conditions can both be characterized by consumers' positive emotional responses, and further research is needed to disentangle emotional valence of the experience.

To summarize, we examined customers' cortical oscillations recorded with the aid of an EEG wireless device during a free in-store navigation. The partial isolation of different sensory fruitions helped us to discuss the results in the light of the presence or deprivation of the ability to touch cosmetic products, thus exploring the role of this sense in consumers' experience.

The performed analysis allowed to determine some main results connected to the presence of specific frequency bands in frontal brain areas when exploring a store with and without the chance to acquire tactile sensory insights. Firstly, a higher presence of Delta and Theta band activity on frontal areas for the non-touch condition was found and interpreted as a need for cognitive control perhaps caused by an incomplete perceptual understanding deriving from the absence of the sense of touch. Secondly, an increase of Beta power for the touch condition was detected in the brain anterior regions, suggesting a cognitive state of sustained attention and enhanced network activity of higher-order somatosensory areas encoding perhaps the sensory aspects of the stimuli. The salience of touch was finally discussed at the light of its evolutionary importance and as a key sense able to provide consumers a complete and coherent perception of their experience.

Despite our study providing novel results exploitable in the sensory marketing field, it also presented some limitations to take into account by future studies. Since our sample size was limited and not balanced for gender variable, it is possible that some gender differences be considered in terms of experiencing the cosmetic store exploration; in one previous study on cosmetics and brain activation, only women were considered (Tanida et al., 2017). Moreover, this study is limited to the cosmetic products field, and no previous studies examined this area of interest *via* EEG technology. To our knowledge, only one study focused on the evaluation of pleasure–displeasure induced by the application of a cosmetic product in terms of cerebral activation exploiting near-infrared spectroscopy (Tanida et al., 2017). In addition, the present study adopted a different EEG methodology compared to the literature on this topic that is mainly based on event-related EEG approach. Therefore, future studies are needed to strengthen both the experimental procedure and present findings.

Overall, the potential of using neuroscientific tools in sensory marketing is still not so widespread, and for this reason, we suggest that future studies could consider the use of EEG wireless device to explore the wider role of touch in consumers' experience in various ecological contexts.

## Data Availability Statement

The datasets generated for this study are available on request to the corresponding author.

## Ethics Statement

The study involving human participants was reviewed and approved by Department of Psychology, Catholic University of the Sacred Heart of Milan, Italy. The participants provided their written informed consent to participate in this study.

## Author Contributions

MB, IV, RS, and LA contributed to the conception and design of the study. MB and LA wrote the first draft and each section of the manuscript. All authors contributed to manuscript revision and read and approved the submitted version.

## Conflict of Interest

The authors declare that the research was conducted in the absence of any commercial or financial relationships that could be construed as a potential conflict of interest.
